# Development and Consumer Acceptability of Functional Bread Formulations Enriched with Extruded Avocado Seed Flour: Nutritional and Technological Properties

**DOI:** 10.3390/foods14244282

**Published:** 2025-12-12

**Authors:** Jesús Salvador Jaramillo-De la Garza, Dariana Graciela Rodríguez-Sánchez, Carmen Hernández-Brenes, Erick Heredia-Olea

**Affiliations:** 1 Escuela de Ingenieria y Ciencias, Tecnologico de Monterrey, Ave. Eugenio Garza Sada 2501, Monterrey 64700, NL, Mexico; sssalvador@tec.mx (J.S.J.-D.l.G.); chbrenes@tec.mx (C.H.-B.); 2 Institute for Obesity Research, Tecnologico de Monterrey, Ave. Eugenio Garza Sada 2501, Monterrey 64700, NL, Mexico; dariana@tec.mx

**Keywords:** avocado seed flour valorization, functional ingredients, plant-based by-products, extrusion, shelf life, bread formulation, circular economy

## Abstract

Avocado processing generates seed by-products rich in dietary fiber that can be upcycled into functional ingredients. This study modified and characterized avocado seed flour via extrusion and enzyme-assisted wet-milling, as well as evaluated its use in wheat bread. The flour was fractionated, and fraction 2 (F2) was selected based on techno-functional performance; it was tested in its non-extruded (NEF2) and extruded (EF2) forms. Breads were prepared by replacing 5% of wheat flour with NEF2 and EF2 (NEB and EB, respectively). Compared with NEF2, EF2 had an 81% higher water absorption index (WAI) and an 18% higher oil absorption index (OAI). Extrusion reduced antioxidant activity ~1.6-fold, consistent with an ~85% decrease in acetogenin content, indicating thermo-mechanical degradation of bioactives linked to bitterness. Analyses were conducted in triplicate (*p* < 0.05). By day 3, crumb hardness increased (EB: 9.65 N; NEB: 6.04 N; control: 5.49 N). In a test with 106 consumers, aroma scores improved for NEB (8.00, IQR 7.00–8.00) and EB (7.00, IQR 5.00–8.00) versus the control (6.00, IQR 4.00–7.00), while overall acceptability, texture, color, and appearance did not differ. These results support EF2 as a functional upcycled ingredient that enhances hydration and aroma, reduces bitterness, and maintains consumer acceptance, aligning with circular economy and clean-label goals.

## 1. Introduction

The industrial processing of avocados generates considerable amounts of by-products, particularly seeds and peels, which together account for up to one-third of the total fruit weight. With global avocado production reaching approximately 9 million tons in 2022, this presents 20–40% of residues, highlighting the importance of developing valorization strategies for these materials [1,2]. Avocado seeds are specially rich in starch content (~60%), dietary fiber (~30–40%), and phenolic compounds, including catechins, procyanidins, and hydroxybenzoic acids [3,4], representing a promising non-traditional source of macromolecules for food applications [1,2]. Their recovery and revalorization align with circular bioeconomy frameworks, helping to mitigate the economic and environmental burdens associated with disposal, while enabling the development of functional food ingredients [5,6].

Recent studies have demonstrated that the incorporation of agroindustrial by-products derived from fruits, nuts, and vegetables into bakery formulations can enhance both nutritional quality and technological functionality [7,8]. Examples of reformulation of bakery products with fiber rich by-products, such as apple pomace, pecan shell, and olive pomace, confirm their potential for sustainable product development without compromising consumer acceptance [9,10]. In this context, the use of avocado seed flour has been evaluated in bakery, beverage, and snack formulations, mainly to enhance fiber content and antioxidant properties [1,2,11]. However, its applications remain limited due to sensory challenges such as bitterness, changes in color, reduced product expansion, presence of fungicidal acetogenins such as persin, as well as partial digestibility of its starch–fiber matrix [4,11,12]. These factors highlight the need for processing strategies that improve the techno-functional and sensory quality of the seed while ensuring its chemical and toxicological safety for food use.

Extrusion is a thermomechanical process that combines high temperature, pressure, and mechanical shear, resulting in structural and molecular changes in starch, proteins, and dietary fibers [13,14]. These transformations include starch gelatinization and dextrinization, protein denaturation, and partial depolymerization or solubilization of dietary fiber, which collectively enhance the water and oil absorption, solubility and phenolic bioaccessibility, while reducing antinutritional compounds [12,15,16,17]. Such modifications are strongly influenced by extrusion parameters, particularly feed moisture, screw speed, and temperature, which determine the physicochemical and techno-functional properties of the resulting flour [13,18]. In bakery applications, these structural changes have shown to improve dough rheology, specific volume, and crumb softness and texture, facilitating the incorporation of fiber- and starch-based ingredients [15,19,20,21]. Other valorization approaches, including fermentation or enzymatic hydrolysis, have also shown partial improvement in starch gelatinization and digestibility or soluble dietary fiber increases but typically only impact one structural component of the matrix, limiting the overall techno-functional improvements [2,18,22,23].

Recent findings indicate that coupling a physical–thermal step such as extrusion with a biochemical process like enzyme-assisted wet-milling can generate synergistic effects, when applied in cereal and legume by-products and in other fruit-derived residues [2,16,18]. This dual strategy improves starch and fiber solubilization, increases bioactive compounds availability and enhances techno-functional properties, while improving sensory quality [22]. Despite promising outcomes in other plant by-products, no previous studies have reported the combined application of extrusion and enzyme-assisted wet-milling for avocado seed flour.

Therefore, integrating extrusion and enzymatic treatment represents a more comprehensive and sustainable approach to avocado seed valorization, overcoming the limitations of single-step processes and creating functional ingredients suitable for bakery applications [5,6,12]. Accordingly, this study investigated extrusion followed by enzyme-assisted wet-milling as a combined strategy to modify and characterize avocado seed flour. Its performance was evaluated in wheat bread at a 5% substitution level, consistent with previous bakery applications using fiber- and starch-rich by-products [7,8,10], by assessing Mixolab behavior, key quality traits, and consumer acceptance.

## 2. Materials and Methods

### 2.1. Materials

All the ingredients used to make the bread were purchased from a local supermarket in Monterrey, Nuevo León, Mexico. These included refined wheat flour, cane sugar, dry yeast, salt, and vegetable shortening. Specifically, refined cane sugar (Avance, Avance Comercial de Monterrey, Monterrey, NL, Mexico), vegetable shortening (Inca, Unilever de México S.A. de C.V., Tultitlán, Edo. de México, Mexico), salt (La Fina, Sales del Istmo, Coatzacoalcos, Veracruz, Mexico), and dry yeast (*Saccharomyces cerevisiae*, Azteca Levadura, Iztapalapa, Mexico) were used. According to the supplier’s technical specifications, the high-gluten refined wheat flour (Espiga, Espiga Harinas, Azcapotzalco, Ciudad de México, Mexico) contained 12.2% moisture, 12.9% crude protein, 0.4% crude fat (ether extract), 0.4% ash, and 74.1% carbohydrates (by difference). Fresh avocado seeds (*Persea americana* Mill., Hass variety), a by-product of guacamole preparation, were also obtained from a local supermarket (HEB, Monterrey, Mexico). Reagents such as sodium hydroxide, hydrochloric acid, nutrient agar (NA), eosin methylene blue (EMB) agar, and potato dextrose agar (PDA) were obtained from Sigma-Aldrich (St. Louis, MO, USA). Alcalase 2.4 L FG was purchased from Novozymes (Bagsvaerd, Denmark), and the total dietary fiber assay kit was purchased from Megazyme (Bray, Ireland). Analytical-grade toluene, chloroform, methanol, and hexane were purchased from CTR Scientific (Monterrey, NL, Mexico). HPLC-grade water and methanol, and HPLC/GC-grade hexane were acquired from Tedia (Fairfield, OH, USA), while sulfuric acid was obtained from DEQ (San Nicolás de los Garza, NL, Mexico). Analytical standards of acetogenins were purified from avocado seed in our laboratory (>97 wt% purity), and their identity was verified by MS-TOF as described by Rodríguez-Sánchez et al. [24]. The standards included: AcO-avocadenyne (1-acetoxy-2,4-dihydroxy-heptadec-12-en-16-yne), AcO-avocadene [(2S,4S)-1-acetoxy-2,4-dihydroxy-n-heptadeca-16-ene], AcO-avocadiene B (1-acetoxy-2,4-dihydroxy-heptadeca-12,16-diene), Persediene [(2R,16E)-1-acetoxy-2-hydroxy-4-oxononadeca-16,18-diene], Persenone C [(2R,5E,16E)-1-acetoxy-2-hydroxy-4-oxononadeca-5,16-diene], Persenone A [(2R,5E,12Z,15Z)-1-acetoxy-2-hydroxy-4-oxoheneicosa-5,12,15-triene], Persin [(2R,12Z,15Z)-1-acetoxy-2-hydroxy-4-oxoheneicosa-12,15-diene], and Persenone B [(5E)-1-acetoxy-2-hydroxy-4-oxononadeca-5-ene].

#### Preparation of Avocado Seed Flour

Approximately 10 kg of clean avocado seeds (5.15% moisture) were coarsely ground using a meat grinder (M-12-FS, Torrey, Guadalupe, NL, Mexico). The ground material was then dried in a convection oven (Electrolux, Stockholm, Sweden) at 55 °C for 16 h to reduce the moisture content and improve its shelf stability. After drying, the material was milled using a knife mill (Wiley Mill Standard Model No. 3, Philadelphia, PA, USA) equipped with a 1 mm mesh to obtain fine flour. The resulting avocado seed flour was vacuum-sealed and stored at 25 ± 2 °C until further processing. This flour was used as the raw material for the subsequent treatments, including extrusion and enzymatic hydrolysis.

### 2.2. Avocado Seed Flour Extrusion

Extrusion was performed in a co-rotating twin-screw extruder (BTSM-30, Bühler AG, Uzwil, Switzerland) equipped with 600 mm screws (L/D 20). The high-shear screw configuration described by Cortés-Ceballos et al. [25] was used; conditions were adapted from cereal-based applications [26] and kept constant across batches. A single barrel temperature setpoint of 130 °C was employed, with a die-plate temperature of 81.0 °C. Solids and water feed rates were 3.5 kg/h and 6.0 kg/h, respectively, at a screw speed of 400 rpm, using a single-orifice circular die of 4 mm. The specific mechanical energy (SME) was 277.4 ± 8.7 Wh/kg, and the torque was 25.97 ± 0.74% (62.93 ± 1.99 N·m), calculated as the average of three independent extrusion runs. After extrusion, samples were dried at 55 °C for 16 h in a convection oven (Electrolux, Stockholm, Sweden), equilibrated to room temperature, and milled through a 1 mm screen using a knife mill (Wiley Mill, Model 3, Philadelphia, PA, USA). Powders were vacuum-sealed and stored under room temperature (25 ± 2 °C). Samples are referred to as NEF (non-extruded flour) and EF (extruded flour). Extrusion was performed on three independent production days under identical operating conditions, resulting in three separate batches considered as experimental replicates.

### 2.3. Enzyme-Assisted Wet-Milling

A total of 320 g of NEF and EF were suspended in 1.6 L of distilled water in a 2 L bioreactor (Sartorius). Protease (Alcalase 2.4 L FG) was added at a ratio of 1 mL of enzyme per 50 g of flour (1:50), then the mixture was incubated for 2 h at pH 8.0, 40 °C and 150 rpm, as described by Xiao et al. [27]. The resulting hydrolysates were processed for 1 min in a commercial blender and then passed through a No. 80 mesh sieve. The portion that was retained (fraction 1) was blended again for 30 s and sieved once more. All the material that passed through the sieve was collected and centrifuged for 5 min at 3000 rpm (~805× *g*). The resulting pellet was identified as fraction 2, and the supernatant as fraction 3. All fractions were dried at 55 °C in a convection oven (Electrolux, Stockholm, Sweden) until constant weight was achieved. The average drying time was 19.7 ± 3.6 h, primarily influenced by the initial sample mass. For bread formulations, only fraction 2 was selected due to its higher yield compared to fractions 1 and 3, and its suitability for incorporation into dough, as determined by preliminary trials. The enzyme-assisted wet-milling and fractionation were carried out in multiple independent batches (six for NEF and four for EF) and the results are expressed as mean ± standard deviation across those replicates.

### 2.4. Proximate Composition

The proximate composition of NEF, EF, and bread samples was analyzed to quantify key nutritional components following standardized procedures. Moisture content, total dietary fiber (TDF), insoluble dietary fiber (IDF), and soluble dietary fiber (SDF) were quantified using AACC Methods 32-05.01 and 32-07.01 [28]. Ash content was determined by incinerating 1 g of sample in a muffle furnace (Barnstead Thermolyne, Model FA7915, Dubuque, IA, USA) at 550 °C for 5 h according to AOAC Method 923.03 [29]. Crude fat was quantified by Soxhlet extraction using petroleum ether as the solvent (AOAC Method 920.85), and crude protein was determined by the Kjeldahl method with a nitrogen-to-protein conversion factor of 6.25 (AOAC Method 978.02). Carbohydrate content was calculated by difference as described by Adebiyi et al. [30]. All determinations were carried out in triplicate, except for dietary fiber measurements, which were conducted in quadruplicate. Analyses were performed as technical replicates using subsamples from each bread and flour type.

### 2.5. Acetogenins Content

Acetogenins were extracted from NEF and EF flours obtained as described in Section 2.2. The extraction procedure followed the method reported by Rodríguez-López et al. [31], with minor modifications. Briefly, 2 g of flour were mixed with 15 mL of acetone and homogenized using a Polytron homogenizer (Ultra-Turrax T25, IKA-Werke, Staufen im Breisgau, Germany) at 16,000 rpm for 3 min. The mixture was sonicated for 1 min in an ultrasonic bath (Model 50T, VWR, Radnor, PA, USA) and shaken for 15 min at room temperature (25 ± 2 °C) using a multi-shaker (Lab-Line Incubator-Shaker, Thermo Fisher Scientific, Chicago, IL, USA). After centrifugation at 10,564 rpm (~10,000× *g*) for 10 min, the supernatant (organic extract) was dried under nitrogen. The residue was re-dissolved in 2 mL of hexane and 2 mL of deionized water, vortexed, and centrifuged at 5000× *g* for 5 min to separate the phases. The organic layer was collected, dried under nitrogen, re-suspended in HPLC-grade isopropanol (1 mg/mL), and filtered through a 0.45 µm PTFE membrane prior to chromatographic analysis.

The identification and quantification of acetogenins were performed on an Agilent 1260 Infinity HPLC system (Santa Clara, CA, USA) coupled to a G4212B photodiode array (PDA) detector, following the method reported by Rodríguez-Sánchez et al. [24]. The PDA detector was set at 208 nm for Persin and Persenone B, and at 220 nm for the remaining acetogenins. Quantification was carried out using the external standards method, based on a five-point calibration curve for each available standard. Chemical structures of quantified acetogenins are presented in Table S1.

### 2.6. Antioxidant Activity

The antioxidant capacity of NEF and EF was determined using the DPPH radical scavenging assay, as described by Villasante et al. [32]. A DPPH standard solution was prepared and mixed with the test samples. The absorbance was measured at 515 nm after 60 min of incubation at 37 °C using a Fluostar Omega UV-Vis microplate spectrophotometer (Paris, France). The radical scavenging activity was expressed as the percentage of DPPH inhibition. Each determination was performed in triplicate as technical replicates using homogenized flour samples from each treatment.

### 2.7. Water Absorption Index (WAI) and Oil Absorption Index (OAI)

The water absorption index (WAI) and oil absorption index (OAI) of the different fractions (NEF and EF) were determined according to Serna-Saldívar [33]. All measurements were performed in triplicate. Analyses were carried out as technical replicates using homogenized flour samples. The indices were calculated using Equations (1) and (2), respectively:

(1) $$WAI\;\left(g/g\right)=Weight\;of\;hydrated\;sediment\;\left(g\right)/Dry\;weight\;of\;sample\;\left(g\right)$$

(2) $$OAI\;\left(g/g\right)=Weight\;of\;oil\;retained\;sediment\;\left(g\right)/Dry\;weight\;of\;sample\;\left(g\right)\;$$

### 2.8. Bread Making Procedure

Bread preparation followed the method described by Acosta-Estrada et al. [34], with minor modifications to adapt the composite flour formulations. Bread formulations expressed in 100 g flour basis are shown in Table 1.

The breadmaking procedure included the following steps:

1. Ingredients were mixed and kneaded until a homogeneous dough was obtained (KitchenAid, 5K45SS, Elkgrove Village, St. Joseph, MO, USA).
2. The dough was fermented at 30 °C and 85% relative humidity for 60 min in a fermentation cabinet (National Manufacturing Co., Lincoln, NE, USA).
3. Moulded loaves were proofed under the same conditions for 40 min.
4. Baking was carried out at 180 °C for 20 min in a convection oven (Electrolux, Stockholm, Sweden).
5. Baked loaves were cooled at 25 ± 2 °C for 1 h.
6. Samples were packaged in new resealable plastic bags made of food-grade polyethylene to prevent moisture loss and microbial contamination.
7. Packaged breads were stored at ambient temperature (25 ± 2 °C) until further analyses.

Slices from each bread (10 g) type were used for sensory and microbiological evaluations. All analyses performed on bread samples were carried out in triplicate as technical replicates using subsamples taken from the same production batch.

### 2.9. Bread Characterization

#### 2.9.1. Mixolab Analysis

The rheological behavior of NEF and EF was evaluated using a Mixolab device (Chopin, Tripette et Renaud, Paris, France), following the ICC Standard Method No. 173 [28], as described by Villasante et al. [8]. Each determination was performed in triplicate as technical replicates using composite flour samples from each treatment.

#### 2.9.2. Specific Volume and Density

The loaf volume was determined by the rapeseed displacement method following AACC Method 10-05.01 [28] at the storage of 0 h. Specific volume (cm^3^/g) was calculated using Equation (3).

(3) SV=V/m 

where *SV* is the specific volume of bread (cm^3^/g), *V* is the loaf volume (cm^3^) and *m* is the loaf mass (g).

Density (g/cm^3^) was calculated as the reciprocal of specific volume, as shown in Equation (4) [35]:

(4) ρ=m/V 

where ρ is the bread density (g/cm^3^). All measurements were performed in triplicate as technical replicates using loaves taken from the same production batch.

#### 2.9.3. pH and Total Titratable Acidity (TTA)

Ten grams of each bread sample were homogenized with 100 mL of distilled water and blended for 5 min. The pH was measured using a calibrated potentiometer (Crisol GLP 22, Crison Instruments, S.A., Alella, Barcelona, Spain) with an electrode (Crisol 52 31, Crison Instruments, S.A., Alella, Barcelona, Spain) at room temperature (25 ± 2 °C). TTA was determined by homogenizing 10 g of each sample with 90 mL of distilled water using an Ultra-turrax homogenizer (IKA-Werke, Staufen im Breisgau, Germany). Titration was performed with 0.1 M NaOH, and results were expressed as mL NaOH/10 g of sample. All determinations were carried out in triplicate as technical replicates using subsamples from the same batch of bread.

#### 2.9.4. Water Activity (Aw)

Five grams of each bread sample were sealed in measurement capsules and analyzed using an Aqualab CX-2 device (Decagon Devices, Inc., Pullman, WA, USA) at 20 °C. Water activity was measured in triplicate and used as a predictor of microbial growth risk and overall bread stability.

#### 2.9.5. Texture Profile Analysis (TPA)

TPA was conducted using a TVT 6700 Texture Analyzer (Perten Instruments, Hägersten, Sweden) which was equipped with a 45 mm diameter aluminum probe. Slices (15 mm thick) from the center of each loaf were compressed twice following AACC Method 74-10.02 [28]. The test was configured with a pre-test speed of 1 mm/s, a test speed of 2 mm/s, a post-test speed of 10 mm/s, 40% strain, and trigger force of 5× *g*. The parameters assessed included hardness, springiness, cohesiveness, chewiness, resilience, and adhesiveness. Data was processed using the TexCalc 5 software. Measurements were performed on day 0, day 1, and day 3 post-baking, with five technical replicates per treatment and time point.

#### 2.9.6. Consumer Sensory Evaluation

Consumer acceptance test was conducted one day after baking to evaluate the sensory quality of the bread samples. The panel consisted of 106 untrained consumers (57 females and 49 males) aged 18–46 years, all of whom were students or collaborators from Tecnológico de Monterrey who were frequent bread consumers. Consumers were excluded if they were pregnant or reported food allergies. Samples (10 g each of CNB, NEB, and EB), including crumb and crust were served in three-digit randomly coded plastic cups, covered to prevent moisture loss, and presented in randomized order under controlled laboratory conditions (25 ± 2 °C, neutral lighting). Participants were provided with unsalted crackers and room-temperature water to cleanse the palate between samples and were instructed not to eat, drink, or smoke for at least one hour prior to testing. Consumers were asked to rate sensory characteristics of each bread sample, including texture, flavor, aroma, appearance, crumb color and overall liking using a 9-point hedonic scale (1 = dislike extremely, 2 = dislike very much, 3 = dislike moderately, 4 = dislike slightly, 5 = neither like nor dislike, 6 = like slightly, 7 = like moderately, 8 = like very much, 9 = like extremely). Each evaluation was recorded on an individual score sheet designed for hedonic testing. The sensory session lasted approximately 30 min. All participants provided written informed consent prior to participation, and the study protocol was reviewed and approved by the Institutional Ethics Committee of Tecnológico de Monterrey (approval number: CSER-DBT-251022).

### 2.10. Statistical Analysis

Unless otherwise stated, all measurements were performed in triplicate as technical replicates. Data independence, normality, and variance homogeneity were verified prior to analysis using the Shapiro–Wilk and Levene’s tests, respectively. Parametric data were analyzed by Student’s *t*-test or one-way ANOVA followed by Tukey’s HSD post hoc test, while Welch’s *t*-test was applied for pairwise comparisons when variances were unequal. For sensory evaluation data (*n* = 106 consumers), the Kolmogorov–Smirnov test was used to assess normality, which confirmed a non-parametric distribution. Therefore, sensory scores were analyzed using the Kruskal–Wallis test followed by pairwise Mann–Whitney tests with Bonferroni correction. Statistical analyses were conducted using Minitab 22.2.1 (Minitab Inc., State College, PA, USA), and the significance level was set at α = 0.05.

## 3. Results

### 3.1. Avocado Seed Flour Characterization

#### 3.1.1. Yields of Avocado Seed Enzyme-Assisted Wet-Milling Fractions

Extraction yields of enzyme-assisted wet-milling fractions were calculated before and after extrusion (Table 2). The NEF fraction 1 (NEF1) and fraction 2 (NEF2) yielded 40.24% and 44.73%, respectively (dry-weight basis). These values differed from those reported by Rivera-González et al. [36], who obtained 75.26% and 27.28%, respectively, using a conventional wet-milling process of avocado seed. In their study, fresh seeds were used without prior drying or extrusion pretreatment, under a 1% sodium bisulfite suspension (pH 4.5, 12 h at 25 °C). These distinct process conditions, particularly the higher initial moisture and the absence of thermomechanical stress, likely explain the higher extraction efficiency observed in their results. Compared with the non-extruded samples, extrusion significantly reduced (*p* < 0.05) the extraction yield of fraction 1 (from 40.24% to 28.05%), had no significant effect (*p* > 0.05) on fraction 2 (44.73% to 42.97%), and significantly increased (*p* < 0.05) fraction 3 yield (15.03% to 28.98%). These yield variations may be related to the high mechanical and thermal stress applied during extrusion (SME ≈ 277 Wh/kg; barrel temperature 130 °C). Such processing conditions are known to modify dietary fiber, increasing its solubility [37,38]. Furthermore, other authors have reported that extrusion can promote fiber–starch–protein interactions, which hinder their separation [36,39]. This phenomenon occurs because the combined effects of heat, pressure, and shear induce starch gelatinization and protein denaturation, exposing hydroxyl, carboxyl, and amino groups capable of forming hydrogen bonds and initial Maillard-type linkages with dietary fiber components. These macromolecular associations decrease solubility and extraction efficiency by generating a compact, cross-linked network that limits the recovery of individual fractions [40,41,42]. Conversely, the yield of fraction 2 did not significantly differ from its non-extruded counterpart, confirming that extrusion mainly affects fractions rich in insoluble materials.

#### 3.1.2. Proximate Composition of Avocado Seed Flour

The proximate compositions of NEF and EF avocado seed flour and their enzyme-assisted wet-milling fractions are shown in Table 3. EF had a significantly higher protein content (5.00%) than NEF (4.51) (*p* < 0.05). Furthermore, the enzyme-assisted wet-milling fractions obtained after extrusion showed contrasting behaviors: EF1 and EF2 exhibited the highest protein levels (5.98 ± 0.11% and 6.12 ± 0.11%, respectively), whereas EF3 presented the lowest value (2.03 ± 0.11%). Interestingly, the protein content of EF1 and EF2 was significantly higher (*p* < 0.05) than that of their non-extruded counterparts, NEF1 (4.18%) and NEF2 (3.07%). These findings suggest that extrusion coupled with enzyme-assisted wet-milling may result in either protein enrichment (EF1–EF2) or protein reduction (EF3), depending on the properties of the recovered fraction. In agreement, previous studies have reported that alcalase treatment performed to extruded flour led to higher extraction of proteins since extrusion denatures and increase protein digestibility, increasing their susceptibility to be hydrolysed by alcalase and enhancing solubility in aqueous solutions [16]. During extrusion, protein unfolding and aggregation occur through heat-induced disruption of hydrogen and disulfide bonds, while mechanical shear exposes hydrophobic residues, increasing surface reactivity and subsequent enzymatic accessibility [41,42]. Moderate moisture (20–25%) and pressure (80–100 bar) favor partial denaturation and matrix expansion, promoting extractability, whereas excessive thermal severity (>150 °C) can induce crosslinking and Maillard-type reactions, reducing solubility [40,42]. These mechanisms explain the opposite behaviors observed among EF fractions.

It should be noted that in Table 3, the designation “ND” (Not determined) for dietary fiber in the NEF1–NEF3 and EF1–EF3 fractions indicates that fiber content was not analyzed for these samples. These fractions were obtained through enzyme-assisted wet-milling based on solubility and particle size separation, resulting in compositional heterogeneity that makes gravimetric fiber determination non-representative. Therefore, dietary fiber was quantified only in the whole flours (NEF and EF) to allow a consistent comparison of the overall effect of extrusion and enzymatic treatment on fiber content. In relation to dietary fiber, a slight but non-significant reduction was observed between EF (23.07 ± 0.38%) and NEF (25.54 ± 0.66%). This apparent decrease is likely associated with thermo-mechanical depolymerization and partial solubilization of insoluble polysaccharides occurring during extrusion, as the combined effects of temperature, pressure, and shear can cleave glycosidic linkages and promote the conversion of insoluble dietary fiber (IDF) into soluble dietary fiber (SDF). Such transitions have been reported in chokeberry pomace, where moderate extrusion energy (123–155 °C; 145–222 Wh kg^−1^ specific mechanical energy) enhanced fiber solubility, while higher severity led to re-aggregation or loss of solubilized fractions, producing an apparent decrease in total dietary fiber [43]. Comparable behavior has also been described for wheat bran, where extrusion increased fiber solubility and porosity, improving hydration capacity but reducing gravimetric recovery of total fiber due to structural fragmentation and altered extractability [40]. Consistently, recent studies confirm that the combination of extrusion with enzymatic steps intensifies the disassembly of non-starch polysaccharides. Lewko et al. [42] observed that cellulase–xylanase-assisted extrusion of wheat flour at 40–80 °C and 23–27% moisture significantly reduced the insoluble fiber fraction while increasing hydration and solvent retention capacity, evidencing the breakdown of hemicellulose and arabinoxylan networks. Similarly, Sui et al. [23] reported that high shear and temperature induce microstructural collapse and redistribution of water within plant matrices, facilitating fiber solubilization. Additionally, the subsequent enzyme-assisted wet-milling step may have intensified this effect: enzymatic hydrolysis of partially disrupted cell-wall polymers can further depolymerize hemicellulose and pectin chains, decreasing the measurable insoluble fraction, as reported in hybrid extrusion–enzymatic treatments [44]. Likewise, Li and Li [41] demonstrated that high-temperature extrusion (90–130 °C) promotes glycosidic bond cleavage and exposure of hydrophilic groups in β-glucans, enhancing solubility and supporting the proposed mechanism of thermo-mechanical depolymerization. Together, these findings indicate that the small numerical decrease in EF reflects changes in solubility and extractability rather than an actual loss of dietary-fiber mass.

For ash content, the slight numerical increase in EF (2.66%) compared to NEF (2.47%) (*p* < 0.05) may be related to the redistribution of minerals bound to the fiber matrix, which are released as polysaccharide linkages are cleaved during extrusion [37,42]. Fat content also showed no significant differences between NEF (1.18%) and EF (0.78%) (*p* > 0.05). However, lipid oxidation, formation of protein–lipid complexes, and physical entrapment within the denatured protein–fiber matrix may reduce extractable fat [23]. The combined effect of shear and local heating accelerates oxidation and induces non-covalent interactions, as reported in other extruded plant systems [41]. Carbohydrate content likewise showed no significant differences between NEF and EF (*p* > 0.05), consistent with previous reports [45,46]. Nevertheless, redistribution of soluble carbohydrates among fractions may occur through gelatinization and dextrinization of starch-like components during extrusion, altering apparent recovery [42]. Overall, these compositional trends are consistent with the physicochemical transformations induced by extrusion, where temperature, shear, and moisture collectively govern protein denaturation, lipid oxidation, fiber depolymerization, and mineral redistribution, defining, the process–structure–property relationships of the extruded avocado seed matrix.

#### 3.1.3. Acetogenin Content

As shown in Table 4, extrusion significantly reduced (*p* < 0.05) the concentration of acetogenin in avocado seed flour, with total levels decreasing from 11.99 ± 1.37 mg/g (dry-weight basis) in NEF to 1.83 ± 0.09 mg/g in EF, corresponding to an overall reduction of 84.71%. Among the eight individual acetogenins quantified, reduction ranged from 77.59% (for persin) to 94.41% (for AcO-avocadiene B). Comparable reductions were also observed by Permal et al. [12], who reported a reduction of ~62% in Persin content, the only acetogenin quantified in their study, when avocado seeds were subjected to friction cooking at 130 °C and a feed rate of 5.3 kg/h to produce ready-to-eat extruded snacks.

Previous studies have reported that acetogenins can remain relatively stable under temperatures up to 120 °C for 15 min [47] or 130 °C for 5 min [12], as well as under high-pressure processing conditions (600 MPa for 3 min) [47]. In contrast, the more drastic reductions observed in the present study may be related to the combined effects of thermal energy and shear forces during extrusion. Furthermore, Permal et al. [12] suggested that Persin losses may be related to its interactions with other food matrix components during extrusion.

Structural features may also play an additional role in acetogenin stability. As shown in Table S1, these molecules are lipid derivatives containing 1-acetoxy-2,4-dihydroxy- and 1-acetoxy-2-hydroxy-4-oxo motifs, as well as trans-enone groups. The most stable molecules to extrusion were persin followed by persenones A, B and C (Table 4), all of which possess a trans-enone group at C-4 position. These findings are consistent with the observations of Pacheco et al. [47], who evaluated acetogenin concentration in a model food system during storage (vacuum-sealed packs at 25 °C and 4 °C for 42 days). Furthermore, Rodríguez-Sánchez et al. [48] suggested that trans-enone group in these molecules may facilitate hydrogen donation to adjacent carbon atoms, thereby conferring antioxidant activity and contributing to molecular stabilization.

The reduction in acetogenins concentration may have practical advantages. On the one hand, these compounds have been identified as key contributors to an unpleasant bitter after-taste [49]. On the other hand, toxicological effects have been documented in animal studies, particularly for persin, at doses ranging from 60–200 mg/kg body weight [50,51]. Moreover, since avocado seeds are not traditionally consumed as food ingredients, safety considerations are essential. In this context, the partial removal of acetogenins during extrusion could improve the sensory profile while mitigating potential safety concerns. However, it is important to highlight that persin concentrations reported in avocado pulp (3.5–6.2 mg/g DW), the edible portion of the fruit, are higher than those reported in seeds (1.7–2.8 mg/g DW) [24,31], and comparatively higher than the levels measured in NEF and EF in the present study.

#### 3.1.4. Antioxidant Activity

As observed in Table 5, antioxidant activity (AA), determined by the DPPH• assay significantly decreased (*p* < 0.05) from 53.34 ± 1.63% in NEF to 14.55 ± 3.37% in EF, representing a 3.6-fold reduction. Similarly, Permal et al. [12] observed a 3.7–4.76-fold reduction in AA in avocado seeds that were subjected to friction cooking (130 °C), as determined by FRAP and CUPRAC assays. Subsequent enzyme-assisted wet-milling fractionation further decreased AA. In the non-extruded samples, NEF1 (35.22 ± 1.95%) and NEF2 (30.30 ± 4.52%), AA was not significant different (*p* > 0.05), and NEF3 (23.90 ± 8.99%) displayed the lowest value. A similar trend was observed in fractions from extruded avocado flour, where AA of EF1, EF2 and EF3 decreased progressively to 22.78 ± 4.86%, 18.48 ± 2.33%, and 12.27 ± 0.15%, respectively; however, differences among fractions were not statistically significant (*p* > 0.05). Comparatively, AA in all non-extruded fractions remained higher than their extruded counterparts (*p* < 0.05), suggesting that extrusion was the predominant factor driving AA losses, beyond wet-milling.

Previous studies have consistently shown that the AA of avocado seed is closely related to its pool of bioactive compounds. For instance, Tan et al. [52] reported that total phenolic, flavonoid, and anthocyanin contents exhibit a strong positive correlation with antioxidant activity measured by DPPH and FRAP assays in methanolic extracts. Similarly, Weremfo et al. [53] highlighted the contribution of specific phenolics, particularly rutin, catechin, and syringic acid, to DPPH and ABTS radical scavenging. Moreover, Rodríguez-Sánchez et al. [48] attributed the lipophilic-ORAC activity of purified avocado pulp fractions to seven acetogenins, including persin, persediene, and persenones A and B, all of which have also been identified in avocado seed. In light of this evidence, the marked decrease in antioxidant activity observed in our study after extrusion can be associated with the loss of both phenolic compounds and acetogenins.

Although phenolic content was not quantified in this study, previous reports indicate simultaneous reductions in phenolic content and antioxidant activity after extrusion in other matrices [54,55]. In our case, the significant decrease (*p* < 0.05) in acetogenin content was observed (Table 4), consistent with the reductions in persin reported by Permal et al. [12]. Mechanistically, such decreases may result from thermal degradation of labile compounds, structural rearrangements induced by high temperature and shear, covalent interactions between polyphenols and macromolecules, or the polymerisation of phenols and tannins, which limit the accessibility of hydroxyl groups to participate in redox reactions [21,55,56].

Residual antioxidant activity in extruded avocado seed flour and enzyme-assisted fractions may still provide functional benefits when incorporated into food formulations [57]. However, similar to acetogenins [49], certain phenolic compounds, such as tannins, hydroxybenzoic acids, hydroxycinnamic acids, and catechins, identified by Figueroa et al. [58] in avocado seed, are known contributors to bitter taste, which could compromise product palatability [12]. In this regard, extrusion can potentially reduce bitter compounds such as acetogenins and phenolic compounds, improving the sensory profile of the flour.

#### 3.1.5. WAI and OAI

As shown in Table 5, extrusion significantly affected (*p* < 0.05) the functional behavior of avocado seed flour and its fractions. The WAI markedly increased after extrusion, particularly in EF2 (4.62), representing a 1.8-fold increase relative to its non-extruded counterpart (NEF2: 2.55; *p* < 0.05). Similar enhancements have been reported for extruded cassava, black wheat, and barley flours [35,59,60], where high temperature and shear disrupt starch granules, promote gelatinization, and expose hydrophilic groups that improve water-binding. Additionally, this trend is also consistent with thermo-mechanical depolymerization of starch-like and non-starch polysaccharides, which increases surface area and matrix porosity [40,43]. Additionally, protein unfolding and exposure of polar residues during extrusion can enhance water affinity [41]. In line with previous evidence linking structural expansion with higher water-holding capacity [46,61], the combined effects of starch gelatinization and cell-wall modification, intensified by enzymatic wet-milling, likely explain the increase in WAI. Enzyme-assisted extrusion has been shown to increase hydration and solvent-retention capacity by disassembling hemicellulose–arabinoxylan networks and creating micro-porous structures [42]. EF1 also showed elevated WAI (4.11; *p* < 0.05), suggesting partial disruption of starch–protein interactions and increased porosity, as noted for extruded banana and yam blends [26,62]. Conversely, Fraction 3 exhibited the lowest WAI values, likely due to its limited ability to retain water. For the Oil Absorption Index (OAI), trends varied among fractions: EF2 (2.55) increased (*p* < 0.05), while EF whole flour decreased to 1.70 relative to NEF samples (*p* < 0.05). Such differences agree with reports linking extrusion-induced changes in surface polarity, protein denaturation, and matrix rearrangement to oil-binding variability [19,59,63]. This behavior can be further explained by heat-induced unfolding and aggregation of proteins, which expose hydrophobic residues and modify the topology of the matrix, thus altering lipid-binding capacity [23,41]. Exposure of hydrophobic residues or formation of porous matrices may enhance oil retention, whereas excessive processing can limit accessible binding sites. From a technological standpoint, fractions with higher WAI and OAI values may offer advantages in bakery applications requiring improved moisture retention or fat stabilization, contributing to better dough handling, texture, and shelf-life [60,64]. Overall, the combined evidence supports a process–structure–property relationship in which extrusion temperature, shear, and moisture govern water and oil binding through gelatinization, protein denaturation, and polysaccharide depolymerization [41,42,43].

### 3.2. Bread Characterization

Based on the results of the functional characterization, Fraction 2 was selected for breadmaking due to its enhanced water absorption index (WAI) and favorable oil absorption index (OAI), both desirable properties for improving dough hydration and fat retention. Therefore, NEB and EB breads refer specifically to formulations containing 5% NEF2 or EF2 fractions, respectively.

#### 3.2.1. Mixolab Analysis

The Mixolab parameters are shown in Table 6 (see also Figure S1 for representative torque–time profiles). Significant differences (*p* < 0.05) were found for the initial dough consistency (C1), the minimum torque during heating (C4), and the gelling range (C5–C4), whereas the peak torque (C3) and the thermal parameters showed no statistical variation among samples. The control (CNB) presented the highest initial consistency (1.49 N·m), indicating a stronger gluten network and higher water-binding capacity. Both processed flours (NEF and EF) exhibited lower C1 values (1.35 N·m and 1.29 N·m, respectively), suggesting that fiber inclusion and partial starch–protein disruption reduced dough strength and hydration, as reported for wheat–flaxseed and corn-based systems [19,65].

The absence of significant changes in the maximum torque (C3) or in the thermal parameters (tCs, Tc2, β, C3–C2) indicates that neither enzyme-assisted wet-milling nor extrusion substantially affected starch gelatinization kinetics. This apparent stability may result from compensatory effects between starch damage and the increase in soluble dietary fiber, which limits granule swelling and viscosity development [23,43]. Nonetheless, the EF sample showed higher hot-gel stability (C4 = 2.24 N·m) than NEF (1.94 N·m; *p* < 0.05), suggesting a more cohesive starch–protein network with reduced susceptibility to shear and enzyme attack. Similar trends have been described for extruded wheat bran and orange-peel fiber matrices showing water redistribution and increased bound-water fractions [20,40].

During cooling, both EF and CNB exhibited greater torque increase (C5–C4 ≈ 2 N·m) than NEF (1.64 N·m; *p* < 0.05). The stronger gelling response of EF can be associated with partial gelatinization followed by amylose recrystallization, leading to a denser network [66,67]. Although such behavior indicates a moderate retrogradation tendency, it can also enhance thermal stability and elasticity [41]. These patterns are consistent with the process–structure relationships of fiber-enriched doughs, where thermo-mechanical stress promotes starch–protein crosslinking and reinforces gel strength [8,42].

Overall, extrusion favored matrix stabilization during heating and cooling, whereas enzymatic–wet processing produced a softer, less cohesive system. The limited variability in the pasting stage also suggests that the modified flours can sustain similar gelatinization behavior to the control, which may benefit machinability. These thermal–cooling responses could influence starch retrogradation and product staling, as further discussed in Section 3.2.4.

#### 3.2.2. Physicochemical Properties

**Figure 1:**
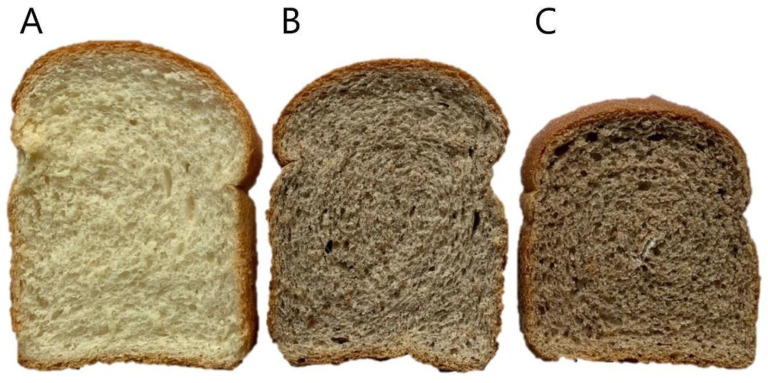
Visual appearance of breads enriched with 5% avocado seed flour (fraction 2). (**A**) Control bread (CNB); (**B**) bread with 5% non-extruded flour (NEB, containing NEF2); (**C**) bread with 5% extruded flour (EB, containing EF2). CNB: control bread; NEB: bread with non-extruded avocado seed flour; EB: bread with extruded avocado seed flour; NEF2: non-extruded avocado seed flour fraction 2; EF2: extruded avocado seed flour fraction 2.

The results for loaf volume, specific volume, and density of the bread samples are presented in Table 7, while their external appearance is shown in Figure 1. Both NEB (non-extruded avocado seed fiber enriched bread) and EB (extruded avocado seed fiber enriched bread) exhibited significantly lower (*p* < 0.05) volume and specific volume values compared to CNB (control bread). Specifically, CNB reached an average loaf volume of 876.43 mL, while NEB and EB showed significantly lower (*p* < 0.05) values (762.50 mL and 715.00 mL, respectively). Similarly, specific volume followed the same trend: 5.81 cm^3^/g for CNB, 5.00 cm^3^/g for NEB, and 4.71 cm^3^/g for EB (*p* < 0.05). In contrast, density increased in breads with avocado seed fiber: 0.17 g/cm^3^ for CNB, 0.20 g/cm^3^ for NEB, and 0.21 g/cm^3^ for EB (*p* < 0.05). These reductions in loaf volume and specific volume, coupled with increased density, may be due to the gluten and starch in the dough being diluted by fiber-rich materials, which weakens the gas-holding matrix during proofing and baking. Similar effects have been reported in formulations incorporating dietary fiber, where gas retention is impaired and crumb structure becomes denser [8,21,68]. Additionally, thermo-mechanical processing during extrusion promotes partial starch gelatinization and disrupts cell-wall integrity, leading to increased water absorption but reduced gas expansion [40,43]. The initial dough consistency (C1), measured using Mixolab, further supports these findings. Both NEF and EF showed lower C1 values than CNB (*p* < 0.05), confirming a reduction in dough viscosity and gluten strength due to fiber inclusion and starch–protein disruption during processing. The slightly lower C1 in EF suggests that extrusion did not increase dough resistance, but rather modified water distribution and matrix plasticity, which may have limited gas retention during proofing. Such effects have been associated with partial starch gelatinization and fiber depolymerization during thermo-mechanical treatments [19,20,43]. Fiber depolymerization and the formation of protein–polysaccharide complexes also increase dough consistency by redistributing bound water and reducing gluten mobility [41,42]. The divergence in performance between NEB and EB may also relate to compositional differences. NEB contained 14.10% protein, while EB had only 12.02%, possibly affecting gluten development and dough elasticity. Furthermore, extrusion-induced microstructural collapse may limit gas cell coalescence, explaining the smaller loaves observed in EB [23]. Despite undergoing extrusion, EB’s lower protein and fiber solubility may have limited its ability to support adequate gas retention, thereby reducing loaf volume. Loaf size remains a critical quality trait influencing consumer acceptance [69]. Practically, adjusting dough hydration, mixing time, or adding vital gluten may mitigate volume losses [7,70].

Additionally, pH, water activity (Aw), and total titratable acidity (TTA) were evaluated to identify potential physicochemical changes associated with fiber enrichment. No significant differences (*p* > 0.05) were observed among CNB, NEB, and EB for any of these parameters (Table 7), indicating that the incorporation of avocado seed fiber, whether extruded or not, did not alter the acid–base balance, buffering capacity, or overall physicochemical stability of the breads.

The Aw values remained within the typical range for fresh bakery products, characteristic of high-moisture matrices that support the growth of spoilage microorganisms such as yeasts and molds, particularly *Penicillium*, *Aspergillus*, and *Rhizopus* species [71]. This stability confirms that moisture distribution and water-binding capacity were not significantly affected by fiber addition. Such behavior reflects the balanced partitioning of bound and free water within the dough matrix, commonly associated with the water-holding capacity of fiber-rich systems [23,40]. Although Aw remained stable, microbial safety during storage still depends largely on hygienic handling and packaging, as post-baking contamination may occur even when Aw is constant [72]. Therefore, maintaining proper storage conditions remains essential to prevent fungal growth and ensure product shelf life.

#### 3.2.3. Proximate Composition of Bread

The proximate composition of the bread samples is presented in Table 8. Significant differences (*p* < 0.05) in protein, fiber, ash, carbohydrate, and moisture contents were observed among formulations. Moisture content ranged from 1.43 ± 0.17% in the control bread (CNB) and 1.46 ± 0.21% in NEB to 1.95 ± 0.16% in EB, indicating that extrusion processing significantly increased (*p* < 0.05) the water retention capacity of the flour. This behavior may be related to structural modifications in starch and fiber matrices that enhance the interaction with water molecules [19]. Thermo-mechanical processing during extrusion induces partial gelatinization of starch and depolymerization of non-starch polysaccharides, which enlarge pore structure and increase hydrophilic site exposure, thereby improving water-binding capacity [40,42]. Higher moisture content in EB could contribute to improved softness and delayed staling, whereas lower values in CNB and NEB are typically associated with firmer crumb structure and faster moisture loss during storage [68]. These results align with previous findings where the inclusion of thermally treated or fiber-rich ingredients modified the water-binding capacity of bakery products [73]. Specifically, the highest protein content was recorded in CNB (15.04%), followed by NEB (14.10%), with significantly lower values in EB (12.02%, *p* < 0.05). This decrease in protein content in fiber-enriched breads may be attributed to the dilution effect caused by the substitution of wheat flour with avocado seed flour, which is naturally lower in protein. Additionally, extrusion can promote partial denaturation and aggregation of proteins, decreasing their solubility and extractability in analytical assays [23,41]. Similar reductions have been reported in fiber-enriched bread formulations with okra flour [73] and extruded quinoa flour [17]. Total dietary fiber (TDF) also followed a decreasing trend: CNB (26.68%), NEB (21.80%), and EB (21.67%). While avocado seed was rich in fiber, its incorporation at relatively low levels (5%) and the modification through extrusion may have affected the measurable TDF values. Extrusion is known to cause fragmentation and solubilization of insoluble dietary fiber (IDF), increasing SDF but occasionally lowering apparent TDF due to enhanced extractability [43,44]. Ash content showed significant differences (*p* < 0.05), with CNB exhibiting the highest value (3.05%), NEB the lowest (2.45%), and EB presenting an intermediate value (2.63%). These results are in line with breads enriched with by-products such as argan press cake and carob pod flour, which typically increase mineral content without compromising acceptability [70,74]. Mineral redistribution during extrusion, associated with the release of ions bound to polysaccharide matrices, can also contribute to these differences [23]. Fat content varied slightly among samples (3.25% in CNB, 3.55% in NEB, and 3.83% in EB), but these differences were not statistically significant (*p* > 0.05). The small variation could be explained by the lipid contribution of the avocado seed flour, particularly in NEB, which retained its native fat fraction. Comparable trends have been observed in bread supplemented with mango peel or pumpkin by-products [75,76]. Thermal processing can also induce limited lipid oxidation and interactions with denatured proteins or fiber networks, affecting extractability rather than total content [23]. Estimated carbohydrate content (calculated by difference) increased in NEB (58.11%) and EB (59.85%) compared to CNB (52.05%) (*p* < 0.05). This trend is consistent with previous reports on the partial replacement of starch-rich ingredients with fruit or vegetable by-products, which may lead to shifts in carbohydrate availability and profile due to the presence of non-starch polysaccharides [68]. These compositional variations collectively confirm the process–structure–property relationship, where extrusion-driven transformations in starch, fiber, and protein modulate hydration, solubility, and nutrient distribution [40,42]. Overall, the observed changes in proximate composition highlight the nutritional impact of incorporating non-extruded and extruded avocado seed fiber into bread formulations. While there is a dilution of protein and fiber at the tested inclusion level, the results remain comparable to other fiber-rich bakery products reported in the literature, supporting the viability of this clean-label ingredient.

#### 3.2.4. Textural Parameters of Bread

The evolution of bread texture during three days of storage is presented in Table 9. On day 0, hardness values were 1.18 N for CNB, 1.36 N for NEB, and 1.32 N for EB, showing no significant differences (*p* > 0.05). Similarly, springiness (0.68–0.74 mm), resilience (0.50–0.53), cohesiveness (0.80–0.88), and chewiness (0.77–0.86 N) did not differ significantly at this stage, indicating that the initial crumb texture was comparable among formulations. By day 1, hardness increased to 3.70 N in NEB and 5.36 N in EB, while CNB remained much softer (1.25 N; *p* < 0.05). This trend intensified on day 3, when EB reached 9.65 N, followed by NEB (6.04 N) and CNB (5.49 N; *p* < 0.05). These results indicate that breads containing extruded avocado seed flour firmed more rapidly during storage. This progressive hardening is consistent with the Mixolab analysis (Section 3.2.1), where the extruded flour exhibited lower initial consistency (C1) but higher gelling torque (C5–C4), suggesting weaker gluten development yet stronger starch retrogradation during cooling. Such behavior favors post-baking firmness due to restricted mobility of amylose and bound water within the crumb matrix [41,66]. Similar phenomena have been reported in breads incorporating extruded or fiber-rich ingredients, where thermally modified starch and soluble fiber fractions create dense polymer networks that limit gas expansion and moisture redistribution [43,69]. The lower protein content in EB likely exacerbated this effect, producing a structure less elastic but more compact. Redistribution of water between bound and free fractions also contributes to reduced plasticization and faster firming during storage [23]. In NEB, the presence of native starch and lipids may have provided partial plasticization, explaining its intermediate firmness and lower elasticity [8]. No significant differences were observed in springiness, cohesiveness, or resilience among samples at any time point, consistent with previous reports for fiber-enriched breads [8]. However, EB showed significantly higher chewiness (*p* < 0.05) by day 3, which could negatively affect sensory perception. This increased chewiness can be attributed to enhanced crosslinking density and partial recrystallization within the fiber–protein matrix [41]. Overall, the incorporation of extruded avocado seed flour modified the bread’s textural behavior during storage, mainly by promoting starch retrogradation and water redistribution rather than by strengthening the gluten network. These findings reinforce the structure–property relationships observed in the Mixolab and proximate analyses, where extrusion enhanced matrix compactness and reduced moisture mobility, leading to a firmer crumb over time.

#### 3.2.5. Consumer Sensory Evaluation

The results of the consumer sensory evaluation, analyzed using non-parametric tests (Kruskal–Wallis and Mann–Whitney with Bonferroni correction), are shown in Table 10. For flavor, CNB and EB obtained the highest scores (*p* < 0.05), and were both significantly superior to NEB. The reduced flavor rating for NEB may be attributed to the presence of native phenolic compounds or residual bitterness in the avocado seed, which tends to diminish during thermal processing. This is consistent with previous reports indicating that non-extruded plant fibers can impart undesirable flavors if not properly processed [65,77]. In contrast, extrusion has been shown to reduce bitter or astringent notes and enhance flavor perception in fiber-enriched products [8]. This improvement can be mechanistically attributed with the exposure of reactive amino and carbonyl groups during extrusion, which favor Maillard-type reactions and precursor formation for desirable aroma compounds [41].

For aroma, NEB and EB achieved significantly higher scores (*p* < 0.05) than CNB. This enhancement can be mechanistically explained by the thermo-mechanical conditions of extrusion and the subsequent baking step, which promote the generation of Maillard-derived volatile compounds (e.g., pyrazines, furans, aldehydes) and thermally modified phenolic derivatives. Bartkiene et al. [78] observed similar increases in pyrazines and furfural in breads containing extruded and fermented bran, while Smith and Peterson [79] reported higher levels of key aroma compounds such as 2-ethyl-3,5-dimethylpyrazine and 2-acetyl-2-thiazoline in extruded whole-grain cereals, supporting the role of extrusion in enhancing precursor availability and volatile formation. According to Tyl et al. [80], extrusion modifies the structure of dietary fiber, increasing soluble fractions and surface reactivity, which facilitates the release of aroma precursors during baking. This is consistent with the increased solubility, porosity, and water-holding capacity reported for extruded matrices, which enhance volatile diffusion and release [40,43]. Redistribution of moisture and a reduction in water activity gradients within the crumb may further modulate the release and perception of volatiles during baking [23]. García-Amezquita et al. [20] also demonstrated that extrusion improves the water-binding and sorption properties of citrus fiber, promoting controlled release of volatiles during heat processing. However, flavor scores did not increase proportionally with aroma, likely due to interactions between dietary fiber and low-molecular-weight taste compounds. Liu et al. [81] showed that thermally modified insoluble fiber exhibits high adsorption capacity for volatiles and taste molecules through chemisorption mechanisms, resulting in moderated flavor perception despite increased aromatic intensity. Similarly, Yu et al. [82] highlighted that extrusion-induced structural rearrangements in dietary fiber alter its interactions with proteins, lipids, and bioactive compounds, thereby affecting the partitioning and perception of flavor-active molecules. Comparable behavior was described by Lewko et al. [42], where enhanced hydration shells and fiber–protein interactions influenced aroma diffusion and release. Despite these differences, consumers did not report any decrease in texture satisfaction, even in EB samples that presented higher hardness and chewiness values (see Section 3.2.4). This aligns with Codină et al. [65], who observed that moderate firmness increases can remain acceptable when accompanied by pleasant aroma and flavor. No significant differences were found among CNB, NEB, and EB in texture, color, appearance, or overall acceptability. All bread samples were microbiologically safe, showing the absence of total coliforms, aerobic mesophilic bacteria, and fungi. In summary, the addition of avocado seed flour, particularly in extruded form, enhanced aroma through the formation of Maillard-derived volatiles and improved precursor availability, while maintaining acceptable flavor, texture, and overall quality. These findings confirm the process–structure–property–perception linkage, where extrusion-induced molecular transformations enhance sensory performance without compromising product safety [41,43]. At a 5% substitution level, avocado seed flour can be considered a viable and safe functional ingredient for bakery applications, combining nutritional and sensory benefits.

## 4. Conclusions

This study represented the first comprehensive report on the combined use of extrusion and enzyme-assisted fractionation to obtain functional avocado seed flours and fractions for bread applications. Extrusion of avocado seed flour (EF) generated fractions with improved techno-functional properties, particularly an increased water absorption index (WAI), while effects on oil absorption capacity (OAI) were fraction-dependent. These modifications reflected structural changes such as enhanced solubility and reactivity of fiber components. Additionally, extrusion significantly reduced acetogenin content and antioxidant activity, but it also mitigated the seed’s inherent bitterness, contributing to improved flavor perception in bread. The fractionation process provided insight into the effect of extrusion on functional and bioactive compounds. Among the obtained fractions, Fraction 2 was selected for breadmaking in both its non-extruded (NEF-F2) and extruded (EF-F2) forms. Importantly, EF-F2 displayed the highest WAI and OAI within the tested fractions, corroborating the functional advantages observed in the analytical assays and confirming its suitability for bakery matrices requiring moisture and fat stabilization. At a 5% substitution level, NEB and EB showed reduced loaf and specific volume compared with the control (CNB), accompanied by increased density and hardness during storage. Despite these effects, sensory evaluation revealed improved aroma perception, with overall acceptability remaining unchanged, demonstrating that the observed technological modifications did not compromise consumer acceptance. All formulations met microbiological safety criteria. From a practical and economic standpoint, this approach demonstrated a feasible pathway for upcycling avocado by-products into value-added food ingredients, contributing to more sustainable and resource-efficient bakery formulations. Taken together, the experimental findings consistently support the conclusions drawn, confirming that the results fully substantiate this study’s objectives. The findings emphasized extrusion as an effective strategy to enhance functionality, reduce undesirable sensory traits, and enable clean-label, nutritionally enriched products. This work was limited by the single substitution level and laboratory-scale processing. Future research should explore higher incorporation levels, pilot-scale validation, and combinations with other by-products or food matrices. Additional studies assessing bioactivity through in vitro digestion or cell-based assays could further elucidate the nutritional potential of these flours. Overall, this study advanced the valorization of agro-industrial residues and provided a replicable framework for reusing plant-based waste streams through extrusion-assisted processes, aligning with circular economy and sustainable food-innovation goals. This work thus provided the first integrated application of extrusion and enzyme-assisted fractionation for avocado seed flour valorization in bakery systems, underscoring its methodological and practical novelty.

## Figures and Tables

**Table 1:** Bread formulations (per 100 g total flour basis) for control (CNB), non-extruded avocado seed flour (NEB), and extruded avocado seed flour (EB) breads.

Ingredients	Sample		
	CNB	NEB	EB
All-purpose wheat flour	100	95	95
NEF2	0	5	0
EF2	0	0	5
Refined cane sugar	6	6	6
Vegetable shortening	3	3	3
Dry yeast	2	2	2
Salt	2	2	2
Water	62	62	62

CNB: control bread prepared with 100% wheat flour; NEB: bread formulated with 5% non-extruded avocado seed flour (fraction 2); EB: bread formulated with 5% extruded avocado seed flour (fraction 2); NEF2: non-extruded avocado seed flour, fraction 2; EF2: extruded avocado seed flour, fraction 2. All formulations are expressed on a 100 g total flour basis (dry weight). Water addition was kept constant across formulations.

**Table 2:** Extraction yields of avocado seed enzyme-assisted wet-milling fractions before and after extrusion (%, dry weight basis).

Sample	Fraction 1				Fraction 2				Fraction 3			
NEF	40.24	±	0.67	^a^	44.73	±	1.16	^a^	15.03	±	0.74	^b^
EF	28.05	±	1.88	^b^	42.97	±	2.73	^a^	28.98	±	0.35	^a^

Values represent mean ± standard deviation (*n* = 6 for NEF, *n* = 4 for EF). Different superscript letters within the same column indicate significant differences (*p* < 0.05) according to Student’s *t*-test (Fractions 1 and 2), or Welch’s *t*-test (Fraction 3). NEF: non-extruded avocado seed flour; EF: extruded avocado seed flour.

**Table 3:** Proximate composition (%, dry-weight basis) of non-extruded (NEF) and extruded (EF) avocado seed flours and their enzyme-assisted wet-milling fractions.

Sample	Parameters																
	Protein (%)				Fat (Q1–Q3)			Ash (Q1–Q3)			Dietary Fiber (%)				Carbohydrates (Q1–Q3)		
Non-extruded and fractionated samples																	
NEF	4.51	±	0.11	^c^	1.18	(1.1061–1.2516)	*^ns^*	2.47	(2.43637–2.53683)	*^ns^*	25.54	±	0.66	^a^	66.31	(65.86–66.76)	*^ns^*
NEF1	4.77	±	0.11	^bc^	1.50	(1.4472–1.5429)	*^ns^*	1.76	(1.75750–1.76283)	*^ns^*	ND				92.01	(91.87–92.15)	*^ns^*
NEF2	4.18	±	0.00	^d^	2.57	(1.6142–3.5191)	*^ns^*	1.04	(0.95691–1.18625)	*^ns^*	ND				92.15	(91.27–93.02)	*^ns^*
NEF3	3.07	±	0.12	^e^	2.81	(0.7255–4.8878)	*^ns^*	12.01	(11.54210–12.36370)	*^ns^*	ND				82.14	(80.37–83.91)	*^ns^*
Extruded and fractionated samples																	
EF	5.00	±	0.11	^b^	0.78	(0.7647–0.7935)	*^ns^*	2.66	(2.64737–2.68217)	*^ns^*	23.07	±	0.38	^a^	68.42	(68.14–68.69)	*^ns^*
EF1	5.98	±	0.11	^a^	0.77	(0.6819–0.8485)	*^ns^*	1.58	(1.41844–1.74978)	*^ns^*	ND				91.71	(91.55–91.86)	*^ns^*
EF2	6.12	±	0.11	^a^	0.64	(0.6232–0.6658)	*^ns^*	1.76	(1.55311–1.96778)	*^ns^*	ND				91.42	(91.23–91.60)	*^ns^*
EF3	2.03	±	0.11	^f^	0.12	(0.1192–0.1261)	*^ns^*	9.61	(8.95317–10.2587)	*^ns^*	ND				88.18	(87.52–88.84)	*^ns^*

Values represent mean ± standard deviation for parametric data or median (Q1–Q3) for nonparametric data (*n* = 3). Different lowercase letters in the same column indicate significant differences (*p* < 0.05) between samples, according to Student’s *t*-test or one-way ANOVA followed by Tukey’s HSD test for parametric data, and the Kruskal–Wallis test for nonparametric data. Carbohydrates were calculated by difference. ND: not determined; *ns*: not significant; NEF: non-extruded avocado seed flour; EF: extruded avocado seed flour; NEF1–3: fractions 1–3 of non-extruded avocado seed flour; EF1–3: fractions 1–3 of extruded avocado seed flour.

**Table 4:** Individual and total acetogenin concentrations (mg/g, dry weight basis) in non-extruded (NEF) and extruded (EF) avocado seed flours.

Acetogenin	Sample								
	NEF				EF				Acetogenin Reduction (%)
AcO-avocadenyne	0.797	±	0.094	^a^	0.059	±	0.002	^b^	92.62
AcO-avocadene	2.939	±	0.301	^a^	0.392	±	0.010	^b^	86.64
AcO-avocadiene B	0.284	±	0.107	^a^	0.016	±	0.001	^b^	94.41
Persediene	0.247	±	0.025	^a^	0.023	±	0.002	^b^	90.53
Persenone C	0.733	±	0.082	^a^	0.083	±	0.002	^b^	88.68
Persenone A	3.109	±	0.362	^a^	0.487	±	0.014	^b^	84.35
Persin	2.561	±	0.179	^a^	0.574	±	0.039	^b^	77.59
Persenone B	1.324	±	0.226	^a^	0.200	±	0.026	^b^	84.88
Total acetogenins	11.994	±	1.375	^a^	1.834	±	0.094	^b^	84.71

Values represent mean ± standard deviation (*n* = 3). Different lowercase letters within the same row indicate significant differences (*p* < 0.05) according to Student’s *t*-test. NEF: non-extruded avocado seed flour; EF: extruded avocado seed flour.

**Table 5:** Antioxidant activity (AA), water absorption index (WAI), and oil absorption index (OAI) of non-extruded (NEF), extruded (EF) avocado seed flours and their enzyme-assisted wet-milling fractions (dry-weight basis).

Sample	AA (%)				WAI (g/g)				OAI (g/g)			
Non-extruded and fractionated samples												
NEF	53.34	±	1.63	^a^	2.87	±	0.01	^d^	2.12	±	0.01	^d^
NEF1	35.32	±	1.95	^ab^	3.57	±	0.03	^c^	2.31	±	0.06	^b^
NEF2	30.30	±	4.52	^ab^	2.55	±	0.01	^e^	2.16	±	0.01	^cd^
NEF3	23.90	±	8.99	^b^	0.50	±	0.02	^g^	1.95	±	0.02	^e^
Extruded and fractionated samples												
EF	14.55	±	3.37	^b^	4.09	±	0.03	^b^	1.70	±	0.00	^f^
EF1	22.78	±	4.86	^b^	4.11	±	0.02	^b^	2.27	±	0.00	^bc^
EF2	18.48	±	2.33	^b^	4.62	±	0.07	^a^	2.55	±	0.03	^a^
EF3	12.27	±	0.15	^b^	1.80	±	0.01	^f^	1.89	±	0.00	^e^

Values are expressed as mean ± standard deviation (*n* = 3). Different superscript letters in the same column indicate significant differences (*p* < 0.05) according to Student’s *t*-test for acetogenins and one-way ANOVA followed by Tukey’s HSD test for AA, WAI, and OAI. ND: not determined; NEF: non-extruded avocado seed flour; EF: extruded avocado seed flour; NEF1–3: fractions 1–3 of non-extruded avocado seed flour; EF1–3: fractions 1–3 of extruded avocado seed flour; AA: antioxidant activity; WAI: water absorption index; OAI: oil absorption index.

**Table 6:** Mixolab parameters of wheat control dough and avocado seed flour doughs (non-extruded and extruded).

Parameter	Sample											
	CNB				NEF				EF			
Initial consistency (C1), N·m	1.49	±	0.02	^a^	1.35	±	0.06	^b^	1.29	±	0.06	^b^
Peak torque during heating (C3), N·m	2.89	±	1.23	^a^	1.99	±	0.06	^a^	2.32	±	0.02	^a^
Minimum torque during heating (C4), Nvm	2.14	±	0.10	^a^	1.94	±	0.02	^b^	2.24	±	0.06	^a^
Stability time (tCs), min	8.00	±	0.01	^a^	8.00	±	0.01	^a^	8.00	±	0.01	^a^
Initial pasting temperature (TC2), °C	52.77	±	0.68	^a^	53.17	±	0.12	^a^	53.53	±	0.84	^a^
Gelatinization rate (β)	0.22	±	0.16	^a^	0.22	±	0.02	^a^	0.29	±	0.05	^a^
Starch gelatinization range (C3–C2), N·m	2.05	±	1.24	^a^	1.28	±	0.01	^a^	1.44	±	0.06	^a^
Cooking stability range (C3–C4), N·m	0.75	±	1.17	^a^	0.15	±	0.01	^a^	0.07	±	0.03	^a^
Gelling (C5–C4), N·m	1.94	±	0.06	^a^	1.64	±	0.06	^b^	2.06	±	0.14	^a^

Values are expressed as mean ± standard deviation (*n* = 3 technical replicates). Different lowercase letters within the same row indicate significant differences (*p* < 0.05) according to one-way ANOVA followed by Tukey’s HSD test. CNB: wheat control dough; NEF: non-extruded avocado seed flour; EF: extruded avocado seed flour.

**Table 7:** Physical and chemical properties of control bread (CNB) and breads supplemented with 5% non-extruded (NEB) or extruded (EB) avocado seed flour.

Sample	Volume				Specific Volume				Density				Aw				pH				TTA			
	(cm^3^)				(cm^3^/g)				(g/cm^3^)												(mL NaOH)			
CNB	876.43	±	8.98	^a^	5.81	±	0.05	^a^	0.17	±	0.00	^c^	0.92	±	0.01	^ab^	6.61	±	0.01	^a^	2.90	±	0.10	^a^
NEB	762.50	±	2.50	^b^	5.00	±	0.03	^b^	0.20	±	0.00	^b^	0.91	±	0.01	^b^	6.68	±	0.09	^a^	2.95	±	0.05	^a^
EB	715.00	±	5.00	^b^	4.71	±	0.04	^b^	0.21	±	0.00	^a^	0.94	±	0.00	^a^	6.61	±	0.00	^a^	2.75	±	0.05	^a^

Values are expressed as mean ± standard deviation (*n* = 3). Different lowercase superscript letters in the same column indicate significant differences (*p* < 0.05) according to one-way ANOVA followed by Tukey’s HSD test. CNB: control bread; NEB: bread with non-extruded avocado seed flour; EB: bread with extruded avocado seed flour.

**Table 8:** Proximate composition (% dry-weight basis) of control bread (CNB) and breads supplemented with 5% non-extruded (NEB) or extruded (EB) avocado seed flour.

Sample	Protein (%)				Fat (%)				Ash (%)				Dietary Fiber (%)				Carbohydrates (%)			
CNB	15.04	±	0.37	^a^	3.25	±	0.18	^a^	3.05	±	0.35	^a^	26.68	±	1.60	^a^	52.05	±	0.60	^b^
NEB	14.10	±	0.57	^a^	3.55	±	0.22	^a^	2.45	±	0.19	^b^	21.80	±	0.80	^a^	58.11	±	1.14	^ab^
EB	12.02	±	0.98	^b^	3.83	±	1.43	^a^	2.63	±	0.25	^ab^	21.67	±	1.09	^a^	59.85	±	4.17	^b^

Values represent mean ± standard deviation (*n* = 3). Different lowercase letters in the same column indicate significant differences (*p* < 0.05) between samples, according to one-way ANOVA followed by Tukey’s HSD test. Carbohydrates were calculated by difference. CNB: control bread; NEB: bread with non-extruded avocado seed flour; EB: bread with extruded avocado seed flour.

**Table 9:** Textural properties of control bread (CNB) and breads supplemented with 5% non-extruded (NEB) or extruded (EB) avocado seed flour during three days of storage.

Sample	Hardness (N)												Springiness (mm)											
	Day 0				Day 1				Day 3				Day 0				Day 1				Day 3			
CNB	1.18	±	0.26	^aB^	1.25	±	0.32	^bB^	5.49	±	0.95	^bA^	0.68	±	0.09	^aA^	0.76	±	0.09	^aA^	0.68	±	0.07	^aA^
NEB	1.36	±	0.25	^aB^	3.70	±	0.59	^aAB^	6.04	±	1.05	^bA^	0.74	±	0.07	^aA^	0.82	±	0.06	^aA^	0.68	±	0.08	^aA^
EB	1.32	±	0.25	^aC^	5.36	±	0.44	^aB^	9.65	±	0.59	^aA^	0.73	±	0.12	^aA^	0.76	±	0.09	^aA^	0.83	±	0.03	^aA^
	Resilience (ratio)												Cohesiveness (ratio)											
	Day 0				Day 1				Day 3				Day 0				Day 1				Day 3			
CNB	0.52	±	0.02	^aB^	0.66	±	0.04	^aA^	0.47	±	0.02	^aB^	0.88	±	0.02	^aA^	0.84	±	0.02	^aA^	0.78	±	0.02	^aB^
NEB	0.50	±	0.00	^aA^	0.52	±	0.04	^bA^	0.48	±	0.03	^aA^	0.88	±	0.02	^aA^	0.86	±	0.02	^aAB^	0.80	±	0.00	^aB^
EB	0.53	±	0.07	^aA^	0.52	±	0.02	^bA^	0.43	±	0.02	^aA^	0.80	±	0.10	^aA^	0.84	±	0.02	^aA^	0.75	±	0.02	^aA^
	Chewiness (N)																							
	Day 0				Day 1				Day 3															
CNB	0.79	±	0.25	^aB^	0.82	±	0.24	^bB^	2.90	±	0.57	^bA^												
NEB	0.86	±	0.17	^aA^	2.63	±	0.47	^aAB^	3.46	±	0.70	^bA^												
EB	0.77	±	0.20	^aC^	3.42	±	0.52	^aB^	6.06	±	0.54	^aA^												

Values are expressed as mean ± standard deviation (*n* = 5). Values with the same lowercase letter within a column do not differ significantly between samples, and values with the same uppercase letter within a row do not differ significantly between storage days (*p* < 0.05), according to one-way ANOVA followed by Tukey’s HSD test. CNB: control bread; NEB: bread with non-extruded avocado seed flour; EB: bread with extruded avocado seed flour.

**Table 10:** Sensory attributes [median (Q1–Q3)] of control bread (CNB) and breads supplemented with 5% non-extruded (NEB) or extruded (EB) avocado seed flour.

Sample	Aroma (Q1–Q3)			Flavor (Q1–Q3)			Texture (Q1–Q3)		
CNB	6.00	(4.00–7.00)	^c^	8.00	(6.00–9.00)	^a^	8.00	(6.75–9.00)	^ns^
NEB	8.00	(7.00–8.00)	^a^	7.00	(6.00–8.00)	^b^	7.00	(5.00–8.25)	^ns^
EB	7.00	(5.00–8.00)	^b^	7.00	(6.00–8.00)	^a^	8.00	(7.00–9.00)	^ns^
	Color (Q1–Q3)			Appearance (Q1–Q3)			Overall acceptability (Q1–Q3)		
CNB	8.00	(7.00–9.00)	^ns^	8.00	(7.00–9.00)	^ns^	7.00	(6.75–8.00)	^ns^
NEB	8.00	(7.00–9.00)	^ns^	8.00	(7.00–9.00)	^ns^	7.00	(6.00–8.00)	^ns^
EB	8.00	(6.00–9.00)	^ns^	8.00	(6.00–9.00)	^ns^	7.00	(7.00–8.00)	^ns^

Values are expressed as median (Q1–Q3) (*n* = 106). Different lowercase letters within the same column indicate significant differences (*p* < 0.05) according to the Kruskal–Wallis test followed by pairwise Mann–Whitney tests with Bonferroni correction. ns indicates no significant differences (*p* < 0.05). CNB: control bread; NEB: bread with non-extruded avocado seed flour; EB: bread with extruded avocado seed flour.

## Data Availability

The original contributions presented in this study are included in the article. Further inquiries can be directed to the corresponding author.

## Supplementary Materials

The following supporting information can be downloaded at https://www.mdpi.com/article/10.3390/foods14244282/s1, Table S1: Chemical structures of acetogenins present in avocado seeds (*Persea americana*) as reported by Rodríguez-Sánchez et al. [24]; Figure S1: Mixolab torque–time curves of wheat control dough (CNB) and doughs containing non-extruded (NEF) and extruded (EF) avocado seed flour.
