# Supplementary material for: Development and Consumer Acceptability of Functional Bread Formulations Enriched with Extruded Avocado Seed Flour: Nutritional and Technological Properties
Source: Foods. 2025 Dec 12;14(24):4282. doi: 10.3390/foods14244282 (PMC12732530; doi:10.3390/foods14244282)
Supplement: Supplementary file 1 [file foods-14-04282-s001.zip › foods-3926356-supplementary.pdf]

Table S1. Chemical structures of acetogenins present in avocado seed (*Persea americana*) as reported by Rodríguez-Sánchez et al. [24].

Acetogenin molecule	Structure
AcO -avocadenyne	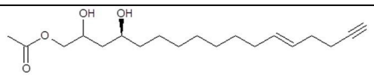
AcO-avocadene	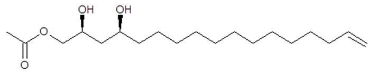
AcO-avocadiene B	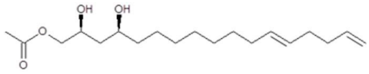
Persediene	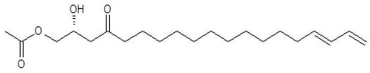
Persenone C	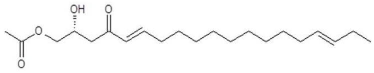
Persenone A	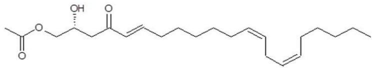
Persin	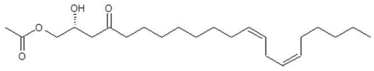
Persenone B	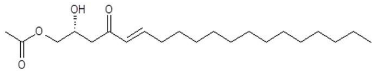

Figure S1. Mixolab torque–time curves of wheat control dough (CNB) and doughs containing non-extruded (NEF) and extruded (EF) avocado seed flour.

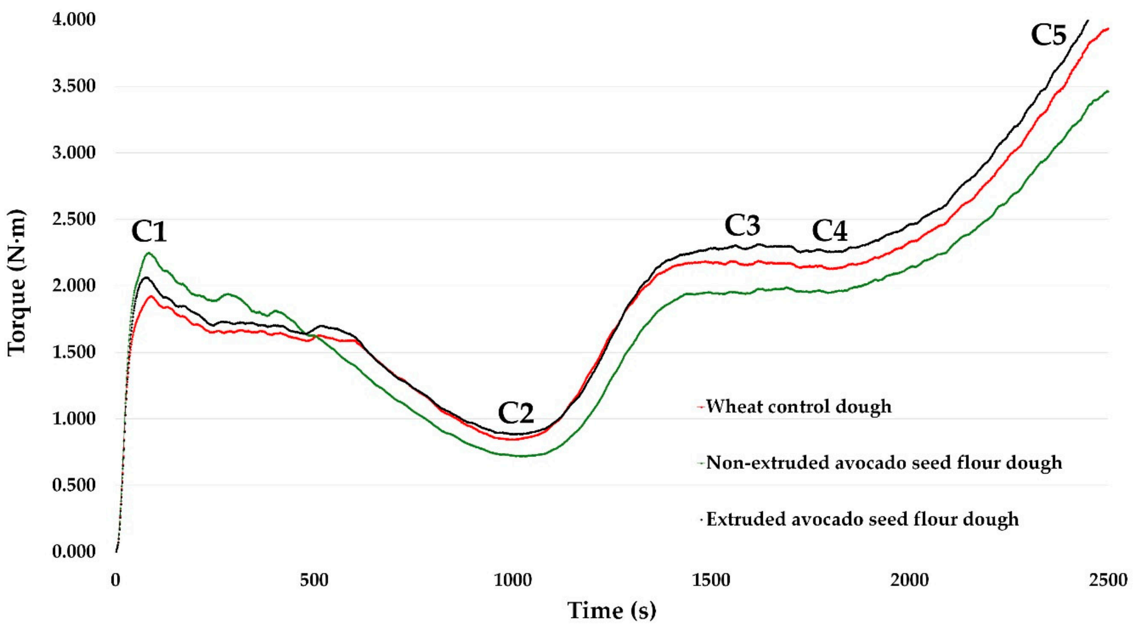

Mixolab torque–time profiles for CNB (wheat control dough, red), NEF (non-extruded avocado seed flour dough, green), and EF (extruded avocado seed flour dough, black). Each curve represents the mean of triplicate measurements. C1: dough development and water absorption; C2: protein weakening; C3: starch gelatinization; C4: hot gel stability; C5: starch retrogradation during cooling. Differences among curves reflect the influence of extrusion on dough consistency and starch behavior.
